# Complete genome sequence of *Kytococcus sedentarius* type strain (**541^T^**)

**DOI:** 10.4056/sigs.761

**Published:** 2009-07-20

**Authors:** David Sims, Thomas Brettin, John C. Detter, Cliff Han, Alla Lapidus, Alex Copeland, Tijana Glavina Del Rio, Matt Nolan, Feng Chen, Susan Lucas, Hope Tice, Jan-Fang Cheng, David Bruce, Lynne Goodwin, Sam Pitluck, Galina Ovchinnikova, Amrita Pati, Natalia Ivanova, Konstantinos Mavrommatis, Amy Chen, Krishna Palaniappan, Patrik D'haeseleer, Patrick Chain, Jim Bristow, Jonathan A. Eisen, Victor Markowitz, Philip Hugenholtz, Susanne Schneider, Markus Göker, Rüdiger Pukall, Nikos C. Kyrpides, Hans-Peter Klenk

**Affiliations:** 1 Los Alamos National Laboratory, Bioscience Division, Los Alamos, New Mexico, USA; 2 DOE Joint Genome Institute, Walnut Creek, California, USA; 3 Biological Data Management and Technology Center, Lawrence Berkeley National Laboratory, Berkeley, California, USA; 4 Lawrence Livermore National Laboratory, Livermore, California, USA; 5 University of California Davis Genome Center, Davis, California, USA; 6 DSMZ - German Collection of Microorganisms and Cell Cultures GmbH, Braunschweig, Germany

**Keywords:** mesophile, free-living, marine, aerobic, opportunistic pathogenic, *Dermacoccaceae*

## Abstract

*Kytococcus sedentarius* (ZoBell and Upham 1944) Stackebrandt *et al.* 1995 is the type strain of the species, and is of phylogenetic interest because of its location in the *Dermacoccaceae*, a poorly studied family within the actinobacterial suborder *Micrococcineae*. *Kytococcus sedentarius* is known for the production of oligoketide antibiotics as well as for its role as an opportunistic pathogen causing valve endocarditis, hemorrhagic pneumonia, and pitted keratolysis. It is strictly aerobic and can only grow when several amino acids are provided in the medium. The strain described in this report is a free-living, nonmotile, Gram-positive bacterium, originally isolated from a marine environment. Here we describe the features of this organism, together with the complete genome sequence, and annotation. This is the first complete genome sequence of a member of the family *Dermacoccaceae* and the 2,785,024 bp long single replicon genome with its 2639 protein-coding and 64 RNA genes is part of the *** G****enomic* *** E****ncyclopedia* of *** B****acteria* and *** A****rchaea * project.

## Introduction

**Figure 1:**
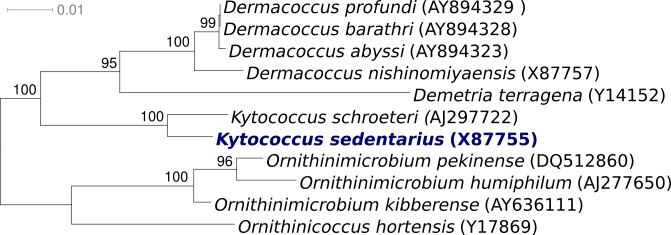
Phylogenetic tree of *Kytococcus sedentarius* strain 541^T^ with all type strains of the family *Dermacoccaceae*, inferred from 1,456 aligned 16S rRNA characters [3] under the maximum likelihood criterion [4,5]. The tree was rooted with four members of the neighboring family *Intrasporangiaceae*. The branches are scaled in terms of the expected number of substitutions per site. Numbers above branches are support values from 1,000 bootstrap replicates. Strains with a genome-sequencing project registered in GOLD [6] are printed in blue; published genomes in bold.

Strain 541^T^ (DSM 20547 = ATCC 14392 = JCM 11482 = CCM 314 and other culture collections) is the type strain of the species *Kytococcus sedentarius*, which is the type species of the genus *Kytococcus* [1]. Strain 541^T^ was first described as *Micrococcus sedentarius* (ZoBell and Upham 1944) [2] and later emended as *Kytococcus sedentarius* in a taxonomic dissection of the genus *Micrococcus* [1]. The organism is of interest for its biotechnological potential as source of natural antibiotics (oligoketides), for its role as an opportunistic pathogen, and for its position in the tree of life, where it represents the scarcely populated genus *Kytococcus* (2 species) within in the actinobacterial family *Dermacoccaceae* [1] (Figure 1). *Kytococcus sedentarius* 541^T^ was first isolated around 1944 from a marine environment [2], but strains of the species were also frequently isolated from human skin [7]. More recently, closely related strains were also isolated from culture-dependant environmental screenings of a non-saline alkaline groundwater environment in Cabeco de Vide in southern Portugal [8], screening for pelagic bacteria in South Korea [9], tropical marine sediments from the intertidal zone off the coast of the Republic Palau [10], from the ciliate Collinia sp.), an endoparasite of euphausiids from the Gulf of California (unpublished literature, GenBank record EU090136), and in a culture-independent analysis of the microbial burden and diversity in commercial airline cabins [11]. Screening of environmental genomic samples and surveys reported at the NCBI BLAST server indicated no closely related phylotypes that can be linked to the species or genus. Here we present a summary classification and a set of features for *Kytococcus sedentarius* strain 541^T^ (Table 1), together with the description of the complete genomic sequencing and annotation.

**Table 1:** Classification and general features of *Kytococcus sedentarius* strain 541^T^ based on MIGS recommendations [12]

**MIGS ID**	**Property**	**Term**	**Evidence code**
	Current classification	Domain *Bacteria*	
		Phylum *Actinobacteria*	
		Class *Actinobacteria*	TAS [13]
		Order *Actinomycetales*	TAS [14]
		Suborder *Micrococcineae*	TAS [13]
		Family *Dermacoccaceae*	TAS [15]
		Genus *Kytococcus*	TAS [1]
		Species *Kytococcus sedentarius*	TAS [1]
		Type strain 541	
	Gram stain	positive	TAS [1]
	Cell shape	spherical, predominantly in tetrads	TAS [1]
	Motility	nonmotile	TAS [1]
	Sporulation	non-sporulating	TAS [1]
	Temperature range	mesophilic	TAS [1]
	Optimum temperature	28-36°C	TAS [1]
	Salinity	nonhalophilic, but growth in media up to 10% (w/v) NaCl	TAS [1]
MIGS-22	Oxygen requirement	mandatory aerobe	TAS [1]
	Carbon source	not reported	
	Energy source	unknown, not starch	NAS
MIGS-6	Habitat	marine	TAS [2]
MIGS-15	Biotic relationship	free-living	NAS
MIGS-14	Pathogenicity	in rare cases	TAS [16,17]
	Biosafety level	1	TAS [18]
	Isolation	slide submerged in sea water	TAS [2]
MIGS-4	Geographic location	probably San Diego	TAS [2]
MIGS-5	Sample collection time	about or before 1944	TAS [2]
MIGS-4.1 MIGS-4.2	Latitude – Longitude	not reported	
MIGS-4.3	Depth	not reported	
MIGS-4.4	Altitude	not reported	

Evidence codes - IDA: Inferred from Direct Assay (first time in publication); TAS: Traceable Author Statement (i.e., a direct report exists in the literature); NAS: Non-traceable Author Statement (i.e., not directly observed for the living, isolated sample, but based on a generally accepted property for the species, or anecdotal evidence). These evidence codes are from the Gene Ontology project [19]. If the evidence code is IDA, then the property was directly observed, for a live isolate by one of the authors, or another expert mentioned in the acknowledgements.

## Classification and features

**Figure 2:**
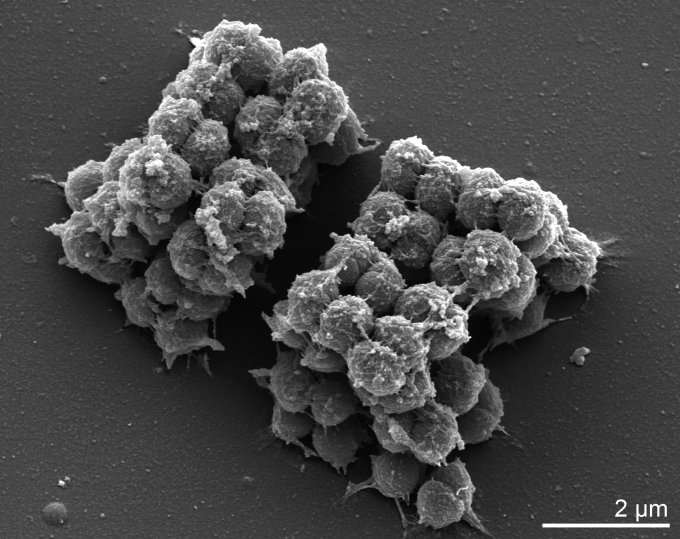
Scanning electron micrograph of *Kytococcus sedentarius* strain 541^T^ (Manfred Rohde, Helmholtz Centre for Infection Biology, Braunschweig)

*Kytococcus sedentarius* cells are spherical/coccoid and occur predominantly in tetrads which can be arranged in cubical packets [1] (Figure 2). Cells are described as Gram-positive, nonmotile, non-encapsulated, and not endospore-forming [1]. *Kytococcus sedentarius* 541^T^ is strictly aerobic and chemoorganotrophic, requires methionine and other amino acids for growth, and grows well in NaCl at concentrations up to 10% (w/v) [1].

*Kytococcus sedentarius* (strain NK0508) is capable of degrading diphenylarsenic acid [20], but not starch [1], and does not produce acids from most carbohydrates and alcohols [1]. Its optimal growth temperature is 28-36°C. Nitrate is reduced to nitrite by some *Kytococcus sedentarius* strains [1]. *Kytococcus sedentarius* is not only described as the source of the oligoketide antibiotics monensin A and B [21], but has also been associated with pitted keratolysis [16], opportunistic infections, and fatal hemorrhagic pneumonia [17].

Figure 1 shows the phylogenetic neighborhood of *Kytococcus sedentarius* strain 541^T^ in a 16S rRNA based tree. Analysis of the 16S rRNA gene copies in the genome of strain 541^T^ differed by one nucleotide from each other, and by up to two nucleotides from the previously published 16S rRNA sequence generated from DSM 20547 (X87755).

### Chemotaxonomy

The murein of *Kytococcus sedentarius* strain 541^T^ contains L-Lys-Glu2, a variation of cell wall type A4α [1]. Mycolic acids and teichonic acids were not reported [1]. Strain 541^T^ contains only completely unsaturated menaquinones with 8-11 isoprene subunits (MK8 to MK11), with MK8 dominating [1]. The major cellular fatty acids are methyl-branched chain iso-C17:1 and anteiso-C17:0, as well as the straight chain saturated C15:0 and C17:0 [1]. Phosphatidylglycerol, diphosphatidylglycerol, and phosphatidylinositol were identified as dominating polar lipids [1]. Reported cytochromes include aa3, c626, c550, b557, b561, and b564 [1].

## Genome sequencing and annotation

### Genome project history

This organism was selected for sequencing on the basis of its phylogenetic position, and is part of the *** G****enomic* *** E****ncyclopedia* of *** B****acteria* and *** A****rchaea * project. The genome project is deposited in the Genome OnLine Database [6] and is deposited in GenBank. Sequencing, finishing and annotation were performed by the DOE Joint Genome Institute (JGI). A summary of the project information is shown in Table 2.

**Table 2:** Genome sequencing project information

**MIGS ID**	**Property**	**Term**
MIGS-31	Finishing quality	Finished
MIGS-28	Libraries used	Two genomic Sanger libraries: 8kb pMCL200 and fosmid pcc1Fos libraries.
MIGS-29	Sequencing platforms	ABI3730
MIGS-31.2	Sequencing coverage	17.3 x Sanger
MIGS-30	Assemblers	phrap
MIGS-32	Gene calling method	Genemark 4.6b, tRNAScan-SE-1.23, infernal 0.81
	Genbank ID	ABUD00000000
	Genbank Date of Release	N/A
	NCBI project ID	21067
	GOLD ID	Gc01042
	Database: IMG-GEBA	2500901761
MIGS-13	Source material identifier	DSM 20547
	Project relevance	Tree of Life, GEBA

### Growth conditions and DNA isolation

*Kytococcus sedentarius* strain 541^T^, DSM20547, was grown in DSMZ medium 92 (3% trypticase soy broth, 0.3% yeast extract) at 30°C. DNA was isolated from 1-1.5 g of cell paste using Qiagen Genomic 500 DNA Kit (Qiagen, Hilden, Germany) with a modified protocol for cell lysis in first freezing for 20 min. (-70°C), then heating 5 min. (98°C), and cooling 15 min to 37°C; adding 1.5 ml lysozyme (standard: 0.3 ml, only), 1.0 ml achromopeptidase, 0.12 ml lysostaphine, 0.12 ml mutanolysine, 1.5 ml proteinase K (standard: 0.5 ml, only), followed by overnight incubation at 35°C.

### Genome sequencing and assembly

The genome was sequenced using a combination of 8 kb and fosmid DNA libraries. All general aspects of library construction and sequencing performed at the JGI website. Draft assemblies were based on 60,742 total reads. The Phred/Phrap-/Consed software package was used for sequence assembly and quality assessment [22-24]. After the shotgun stage, reads were assembled with parallel phrap (High Performance Software, LLC). Possible mis-assemblies were corrected with Dupfinisher [25] or transposon bombing of bridging clones (Epicentre Biotechnologies, Madison, WI). Gaps between contigs were closed by editing in Consed, custom priming, or PCR amplification (Roche Applied Science, Indianapolis, IN). A total of 1,255 additional reactions were necessary to close gaps and to raise the quality of the finished sequence. The completed genome sequence of *Kytococcus sedentarius* 541^T^ contains 61,582 reads. The error rate of the completed genome sequence is less than 1 in 100,000. Together all libraries provided > 17x coverage of the genome.

### Genome annotation

Genes were identified using GeneMark [26] as part of the genome annotation pipeline in the Integrated Microbial Genomes Expert Review (IMG-ER) system [27], followed by a round of manual curation using JGI’s GenePRIMP pipeline. The predicted CDSs were translated and used to search the National Center for Biotechnology Information (NCBI) non-redundant database, UniProt, TIGRFam, Pfam, PRIAM, KEGG, COG, and InterPro databases. The tRNAScanSE tool [28] was used to find tRNA genes, whereas ribosomal RNAs were found by using the tool RNAmmer [29]. Other non-coding RNAs were identified by searching the genome for the Rfam profiles using INFERNAL (v0.81) [30]. Additional gene prediction analysis and manual functional annotation was performed within the Integrated Microbial Genomes (IMG) platform [31].

### Metabolic network analysis

The metabolic Pathway/Genome Database (PGDB) was computationally generated using Pathway Tools software version 12.5 [32] and MetaCyc version 12.5 [33], based on annotated EC numbers and a customized enzyme name mapping file. It has undergone no subsequent manual curation and may contain errors, similar to a Tier 3 BioCyc PGDB [34].

## Genome properties

**Figure 3:**
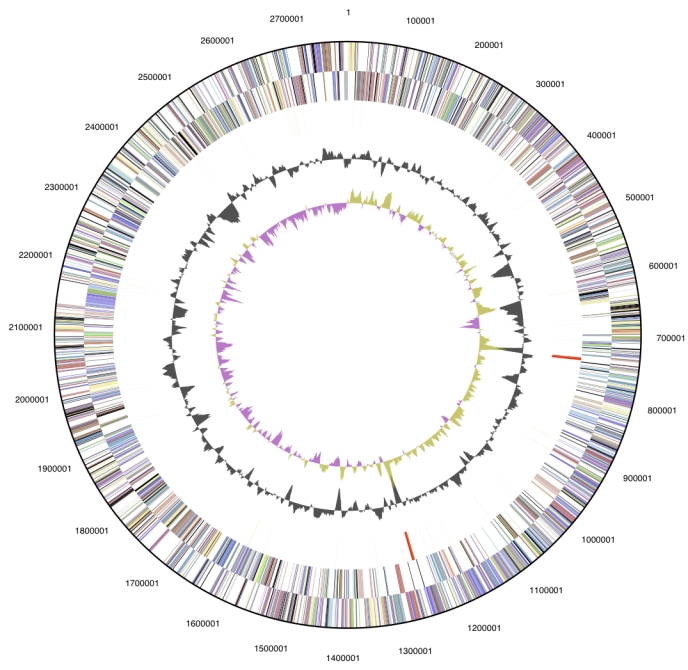
Graphical circular map of the genome. From outside to the center: Genes on forward strand (color by COG categories), Genes on reverse strand (color by COG categories), RNA genes (tRNAs green, rRNAs red, other RNAs black), GC content, GC skew.

The genome is 2,785,024 bp long and comprises one main circular chromosome with a 71.6% GC content (Table 3 and Figure 3). Of the 2,703 genes predicted, 2,639 were protein-coding genes, 64 encoded RNAs. Eighty-four pseudogenes were also identified. In addition, 72.1% of the genes were assigned with a putative function while the remaining ones were annotated as hypothetical proteins.

**Table 3:** Genome Statistics

**Attribute**	**Value**	**% of Total**
Genome size (bp)	2,785,024	
DNA Coding region (bp)	2,558,989	91.88%
DNA G+C content (bp)	1,994,844	71.63%
Number of replicons	1	
Extrachromosomal elements	0	
Total genes	2703	100.00%
RNA genes	64	2.37%
rRNA operons	2	
Protein-coding genes	2639	97.63%
Pseudo genes	84	3.11%
Genes with function prediction	1948	72.07%
Genes in paralog clusters	288	10.65%
Genes assigned to COGs	1851	68.48%
Genes assigned Pfam domains	1908	70.59%
Genes with signal peptides	539	19.94%
Genes with transmembrane helices	595	22.01%
CRISPR repeats	0	0

**Figure 4:**
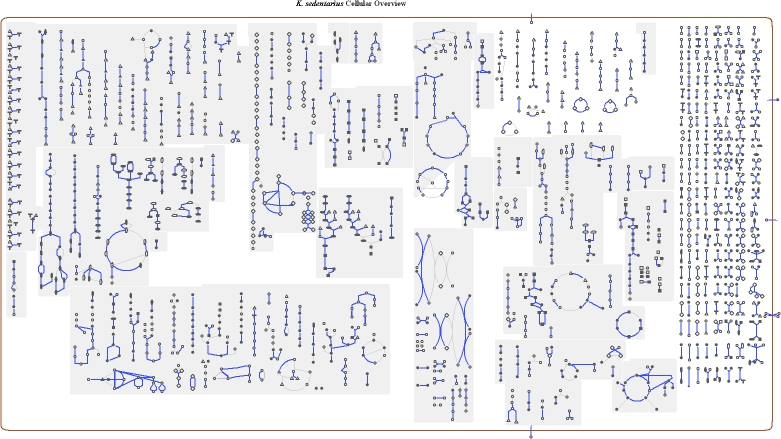
Schematic cellular overview of all pathways of the *Kytococcus sedentarius* strain 541^T^ metabolism. Nodes represent metabolites, with shape indicating class of metabolite. Lines represent reactions.

The distribution of genes into COGs functional categories is presented in Table 4, and a cellular overview diagram is presented in Figure 4, followed by a summary of metabolic network statistics shown in Table 5.

**Table 4:** Number of genes associated with the 21 general COG functional categories

**Code**	**Value**	**%age**	**Description**
J	151	5.7	Translation
A	1	0.0	RNA processing and modification
K	143	5.4	Transcription
L	160	6.1	Replication, recombination and repair
B	2	0.1	Chromatin structure and dynamics
D	22	0.8	Cell cycle control, mitosis and meiosis
Y	0	0.0	Nuclear structure
V	56	2.1	Defense mechanisms
T	73	2.8	Signal transduction mechanisms
M	111	4.2	Cell wall/membrane biogenesis
N	2	0.1	Cell motility
Z	1	0.0	Cytoskeleton
W	0	0.0	Extracellular structures
U	27	1.0	Intracellular trafficking and secretion
O	64	2.4	Posttranslational modification, protein turnover, chaperones
C	99	3.8	Energy production and conversion
G	116	4.4	Carbohydrate transport and metabolism
E	185	7.0	Amino acid transport and metabolism
F	75	2.8	Nucleotide transport and metabolism
H	101	3.8	Coenzyme transport and metabolism
I	86	3.3	Lipid transport and metabolism
P	117	4.4	Inorganic ion transport and metabolism
Q	46	1.7	Secondary metabolites biosynthesis, transport and catabolism
R	229	8.7	General function prediction only
S	160	6.1	Function unknown
-	788	29.9	Not in COGs

**Table 5:** Metabolic Network Statistics

**Attribute**	**Value**
Total genes	2703
Enzymes	531
Enzymatic reactions	922
Metabolic pathways	185
Metabolites	662
